# Applicability and cross-cultural validation of the Chinese version of the Warwick-Edinburgh mental well-being scale in patients with chronic heart failure

**DOI:** 10.1186/s12955-019-1120-2

**Published:** 2019-03-29

**Authors:** Aishu Dong, Xiuxia Zhang, Haitao Zhou, Siyi Chen, Wei Zhao, Minmin Wu, Junyi Guo, Wenjian Guo

**Affiliations:** 1 Emergency Department, the Second Affiliated Hospital of Wenzhou Medical University, College West Road 109, 0577 Wenzhou, Zhejiang People’s Republic of China; 2 Cardiac Department, the Second Affiliated Hospital of Wenzhou Medical University, College West Road 109, 0577 Wenzhou, Zhejiang People’s Republic of China; 3 Chemoradiotherapy Department, the Second Affiliated Hospital of Wenzhou Medical University, College West Road 109, 0577 Wenzhou, Zhejiang People’s Republic of China; 4 Hematology Department, the Second Affiliated Hospital of Wenzhou Medical University, College West Road 109, 0577 Wenzhou, Zhejiang People’s Republic of China

**Keywords:** Validation, Heart failure, Quality of life, Mental health, Patients, Warwick–Edinburgh mental well-being scale, Mental well-being

## Abstract

**Background:**

The mental well-being of patients with chronic heart failure is likely to influence their health-related quality of life and decrease the utilization of public health resources**.** This study assessed the mental well-being of patients with chronic heart failure and evaluated the reliability and validity of the Warwick-Edinburgh Mental Well-Being Scale.

**Methods:**

We conducted a cross-sectional survey from July 2016 to July 2017 among 191 patients with chronic heart failure, and examined psychometric properties of the Warwick-Edinburgh Mental Well-Being Scale, such as internal consistency, reliability, test-retest reliability, and factorial validity of the Chinese version of the Warwick-Edinburgh Mental Well-Being Scale.

**Results:**

One-dimensional construct validity was demonstrated by confirmatory factor analysis. The psychometric properties of the Chinese version of the Warwick-Edinburgh Mental Well-Being Scale were satisfactory in our sample of patients with chronic heart failure. The internal consistency reliability was .948 and the test-retest reliability .925. The item-total correlations ranged from .405 to .872. There was a strong correlation (*r* = .79) between the Chinese version of the Warwick-Edinburgh Mental Well-Being Scale and the five-item World Health Organization Well-Being Index. The Chinese version of the Warwick-Edinburgh Mental Well-Being Scale appears acceptable for use in patients with chronic heart failure, and we were able to verify its reliability and validity with our sample.

**Conclusions:**

The Chinese version of the Warwick-Edinburgh Mental Well-Being Scale is a reliable quantitative tool for evaluating mental well-being in patients with chronic heart failure in clinical settings, and this has important implications for overall assessments of mental well-being in patients with chronic heart failure.

**Electronic supplementary material:**

The online version of this article (10.1186/s12955-019-1120-2) contains supplementary material, which is available to authorized users.

## Background

According to the definition of the World Health Organization, mental health is “a state of well-being in which every individual realizes his or her own potential, can cope with the normal stresses of life, can work productively and fruitfully, and is able to make a contribution to her or his community” [1]. Nowadays, mental health additionally involves the presence of psychological resources, including both eudemonic and hedonic aspects [2, 3]. It has been found that people with better mental health show lower utilization of public health services in some researches [4, 5]. Positive mental health with the definition of “the scientific study of those positive strengths and virtues that enable people & communities to reach optimal levels of health, happiness and well-being” [6], increasingly plays an important role in health-related quality of life, which has gradually been growing interest among people along with economic development, social progress, and the rise in patient-oriented service. It has social consequences and significant health outcomes [7, 8], and it could replace the traditional medical treatment of mental illness [9]. Historically, humans have always been interested in determining the factors that influence their health, well-being, and happiness. A study reported that “the term positive mental health is often used in both policy and academic literature, interchangeably with the term mental well-being” [10]. Mental well-being is a complex construct, covering both affect and psychological functioning with two distinct perspectives: the hedonic perspective, which focuses on the subjective experience of happiness and life satisfaction, and the eudaimonic perspective, focusing on psychological functioning and self realization [3] and has been receiving increasing attention recently, is escalating the government schema [11, 12], and is becoming progressively valued as a key public health indicator. Governments in the worldwide are gradually recognizing the important role of improved mental well-being in aspect of making progress in society, reflecting human capital and mental health promotion initiatives [13]. Especially in Europe, research has increasingly addressed the assessment of mental well-being [5, 14].

Chronic heart failure (CHF) affects physical, psychological, and social aspects in individuals, gradually sets limits on physical activities [15] and leads to psychological and cognitive problems [16], which probably impact their quality of life and well-being. It is reported that more than half of CHF patients live with anxiety, about one third with depression, and nearly half with cognitive impairments [17–19]. However, these outcomes represent only one extreme of the wide-range spectrum of mental health. Whether the CHF patients’ experience with negative aspects of mental health is also associated with mental well-being is still unclear, and there is rarely focusing on the mental well-being.

The Warwick-Edinburgh Mental Well-Being Scale (WEMWBS) was developed to evaluate positive mental health in the students and general population samples [10]. It has been found to be an appropriate instrument for assessing mental well-being in different samples: the general population [20–25]; teachers [26]; adolescents [6, 27, 28]; older people [29, 30]; women with urinary incontinence [31, 32]; people experiencing mental health problems [33, 34]; caregivers of individuals with psychosis [35]; family caregivers of people with dementia [36]. It has been validated in Ireland [27], France [37], Norway [38], Pakistan [39, 40], China [41], Brazil [42], and Spain [43, 44]. The full Chinese version of the WEMWBS has been applied only among undergraduate nursing trainees [41]. Therefore, it is necessary to determine whether the scale may also be used in other populations. There are two versions of WEMWBS- a full version with 14 items and a short version with 7 items of the scale (SWEMWBS). Whether the SWEMWBS shows similar properties to the WEMWBS has yet to be explored [45]. Meanwhile, the short version presents a more restricted definition of mental well-being as it mainly encompasses hedonic items [38]. Therefore, we adopted the 14-item version to monitor mental well-being in CHF patients, as an important virtue of the WEMWBS has been its integrated brief and plentiful description of positive mental well-being.

In this study, we evaluated the psychometric properties of the WEMWBS among CHF patients applying the Chinese version of Warwick-Edinburgh Mental Well-Being Scale (C-WEMWBS) [41]. This was the first time for the C-WEMWBS to be applied in hospitalized CHF patients in China.

## Methods

### Warwick-Edinburgh mental well-being scale

The WEMWBS uses a five-point Likert scale; it contains 14 items, and each item ranges from 1 (none of the time) to 5 (all of the time). The total WEMWBS score ranges from 14 to 70 by totaling the 14 item scores. The higher the overall score, the greater is the level of mental well-being in general population group [10]. The scale assesses psychological functioning (e.g, clear thinking, positive energy, self-acceptance, and competence), positive effect (e.g., cheerfulness, feelings of optimism, and relaxation), and interpersonal relationships [10]. In the United Kingdom, the original WEMWBS has shown good psychometric properties with a single-factor hypothesis, supported by confirmatory factor analysis after allowing for correlation among some of the residuals [10].

The cross-cultural adaptation of WEMWBS into Chinese contained forward and backward translations with assessment of its cultural equivalence, clarity, and initiatory validation which showed high internal consistency reliability, test–retest reliability and preliminary construct validity [41].

### Additional measure

We used an additional measure to test concurrently the validity of the C-WEMWBS. In the present study, all the measures were self-administered. The five-item World Health Organization Well-Being Index (WHO-5) [46] is a scale that evaluates general well-being over the previous 2 weeks. Each item ranges from 0 (at no time) to 5 (all of the time), with the total score ranging from 0 to 25. A total score of under 13 or a score equal to or less than 1 for any item indicates poor well-being or mood problems [47]. The Cronbach alpha was .85 in the current sample.

The present study was carried out in accordance with the ethical standards of the institutional research committee of the Second Affiliated Hospital of Wenzhou Medical University and with the 1964 Declaration of Helsinki and its later amendments or comparable ethical standards.

### Participants

In this study, participants were recruited into the study from a teaching hospital and clinical center in Wenzhou, Zhejiang Province, China with convenience sampling. Patients with primary diagnosis of CHF were included when they met the following criterion: (1) could express themselves clearly, (2) aged over 18, (3) with New York Heart Association (NYHA) [48] functional class II to IV, (4) without addiction to smoking or alcohol, (5) not using antidepressants or anxiolytics, (6) without receiving permanent pacemaker, heart transplant or an implantable cardioverter defibrillator. We excluded the following participants: (1) concurrent inclusion in a study requiring additional visits to research health-care personnel; (2) having received an invasive intervention within the previous 6 months or having one planned during the following 3 months; (3) or ongoing evaluation for heart transplantation.

### Data collection

In this study, we adopted a descriptive, cross-sectional design and aimed to recruit the amount of participants based on a well-accepted minimum of 10 individuals per questionnaire item [43] and less than the maximum of 25 [49]. We collected our data from July 2016 to July 2017. All the participants signed an informed consent form to take part. At the first stage, the structured questionnaire about the patients’ general condition, together with the C-WEMWBS administered by a trained interviewer in a quiet, private room. The participants were asked by a registered physician if they had ever been diagnosed for other diseases, such as diabetes mellitus, hypertension, chronic lung disease, coronary heart disease, arthritis, and stroke, for more than 1 month. Clinical characteristics, such as left ventricular ejection fraction (LVEF) and NYHA classes of the participants, were retrieved from medical records. At the second stage, 35 participants who expressed interest in participating at former stage with health condition expecting steady were selected to evaluate a test-retest reliability analysis. These participants were invited and informed that the repeated questionnaire administration was for a test-retest reliability analysis. The participants completed the 30-item questionnaire with C-WEMWBS that had been embedded in (30-item questionnaire was extracted from the former questionnaire with complete scale of C-WEMWBS to avoid stereotyped image as to capture actual test-retest reliability result) when the patients with CHF visited the outpatient for follow-up in the following 2 weeks.

### Data analysis

We used EpiData 3.1 software (EpiData Association, Odense, Denmark) for double entry and data management. We employed Amos version 17.0 software (Statistics Solutions, Clearwater, FL, USA) and SPSS version 19.0 (IBM Corp., Armonk, NY, USA) for statistical analysis.

We computed descriptive statistics and frequencies for the demographic variables and total scores with the C-WEMWBS. Kolmogorov-Smirnov test was used to check the assumption and normality for the C-WEMWBS scores. We also sought ceiling and floor effects in the response distribution. Associations between C-WEMWBS scores and participant characteristics were evaluated using independent sample *t* tests and one-way analysis of variance. Multiple comparison between the groups was performed using Student-Newman-Keuls method.

We used confirmatory factor analysis and exploratory factor analysis to evaluate the internal structure of the scale. Factor analysis with principal component analysis and varimax rotation were undertaken to assess the construct validity of the C-WEMWBS. Prior to principal component analysis, we evaluated the applicability of the scale with the following standards: a total Kaiser-Meyer-Olkin (KMO) measure of 0.7 or more and a statistically significant Bartlett’s test of sphericity (*P* < .05) correlation coefficient > 0.4 for all variables. The number of components to be retained was determined by eigenvalues > 1, the amount of variance explained, Cattell’s scree plot, reliability analysis, and the interpretability criterion.

We used the Cronbach alpha coefficient to estimate internal consistency, item-total correlations, and reliability [50] of the C-WEMWBS in our sample; a value of .70 or more was considered acceptable [51]. The contents would be included if they had a factor loading of 0.4. Item-total correlations were computed using the Pearson product-moment correlation coefficient. We evaluated test-retest reliability as the interclass correlation coefficient between the first and second administration of the C-WEMWBS.

We calculated both ceiling and floor effects of the 14 items of the scale; the scores were depicted as a histogram, and we inspected the distribution of the scores. We recorded the proportion of individuals with the highest and lowest potential scores. Values of 20% or greater were considered ceiling and floor effects. The values had to be below 20% to guarantee that the 14 items could detect a full range of possible responses in the sample and that response changes over time could be accurately evaluated [52].

Owing to the lack of a gold standard scale for well-being in China, we used the WHO-5 (a Chinese version is available) to estimate the construct validity. We calculated the Pearson correlation coefficient to assess the relationship between the WHO-5 and the C-WEMWBS. A *P* value of <.05 was considered statistically significant.

## Results

### Sample characteristics

In all, 210 patients with CHF were initially invited to take part; of those, 201 agreed to participate, with a response rate with 95.7%(201/210). We excluded from the analyses participants who left more than three unanswered items on each questionnaire (*n* = 10), and the effective rate was 95.0%(191/201). The proportion of missing data for the C-WEMWBS was low (3.0%); none of the items showed a greater likelihood of being left incomplete. The characteristics of the participants were as follows mean age (SD) was 73.79 ± 11.93, 82.2% lived with spouse, 68.6% received elementary education, 57.6% were self-perceived intermediate type of personality. Only 12.5% participants had more than three kinds of comorbidities even though 44.5% had total score of CCI higher than 3, while the percentage of NYHA III was 47.1, and 66.5% had LVEF more than 40%(Table 1, Additional file 1).

**Table 1:** Demographic characteristics of a sample of CHF patients, who completed the C-WEMWBS (*n* = 191)

Characteristics	Variables	Frequency (%)	Mean (SD)	t/F (df)	*P* value
Gender	Male	117(61.3%)	43.37(12.99)	1.551(189)	0.099
	Female	74(38.7)	40.65(9.64)		
Age	≤60	27(14.1%)	50.67(10.86)	14.560(2)	2, 0.000*
	60–80	103(53.9%)	43.17(11.22)		
	≥80	61(31.9)	37.16(10.96)		
Marriage	With spouse	157(82.2)	43.51(11.31)	2.819(44.73)	0.007*
	Without spouse	34(17.8)	36.79(12.86)		
Education level	Elementary education	131(68.6%)	39.92(11.21)	9.693(2)	0.000*
	Secondary education	48(25.1%)	46.85(11.93)		
	College education	12(6.3)	50.25(10.27)		
Comorbidities	None	66(34.6%)	42.88(11.23)	0.620(2)	2,0.539
	1–3	101(52.9%)	41.50(12.35)		
	>3	24(12.5%)	44.08(11.19)		
CCI	1	104(54.5)	44.65(10.79)	3.008(171.135)	0.003
	2	79(44.5)	39.52 (12.50)		
NYHA	II	57(29.8)	46.04(12.02)	5.542(2)	2,0.005
	III	90(47.1)	41.88(11.14)		
	IV	44(23.0)	38.39(11.88)		
Personality	Optimistic	25(13.1)	53.36(9.75)	23.281(2)	2,0.000*
	Pessimistic	56(29.3)	36.05(9.79)		
	Median	110(57.6)	42.99(11.26)		
LVEF	≤40%	64(33.5%)	43.58(13.28)	0.986(108.14)	0.326
	>40	127(66.5%)	41.68(11.06)		

*:*P < 0.01*

### Construct validity

We conducted CFA to test the hypothesized single-factor structure of the C-WEMBWS, and the goodness of fit for a single confirmatory factor model was measured. Assuming no dependencies among the residuals, the initial model showed poor fit (comparative fit index = 0.87, root mean square error of approximation = 0.14). After correlated error terms were added in a stepwise fashion, adequate fit statistics were evident after 15 steps (χ^2^ = 79.04, *df* = 62, *P* = .07; comparative fit index = .99; Tucker-Lewis index = .99; goodness-of-fit index = .95; root mean square residual = .03; root mean square error of approximation = .04), as seen in Table 2. The goodness-of-fit results for a single-factor confirmatory model suggested an adequate one-dimensional structure [53].

**Table 2:** Item-level statistics for responses on the C-WEMWBS in a sample of CHF patients (*n* = 191)

Item	Content	14-item version, no correlated errors	14-item version with correlated errors
C-WEMWBS-1	I’ve been feeling optimistic about the future	2.87	1.128
C-WEMWBS-2	I’ve been feeling useful	2.80	1.106
C-WEMWBS-3	I’ve been feeling relaxed	3.09	.936
C-WEMWBS-4	I’ve been feeling interested in other people	2.60	1.151
C-WEMWBS-5	I’ve had energy to spare	2.23	.998
C-WEMWBS-6	I’ve been dealing with problems well	2.98	1.128
C-WEMWBS-7	I’ve been thinking clearly	3.10	1.071
C-WEMWBS-8	I’ve been feeling good about myself	2.89	1.107
C-WEMWBS-9	I’ve been feeling close to other people	3.92	.923
C-WEMWBS-10	I’ve been feeling confident	2.91	1.772
C-WEMWBS-11	I’ve been able to make up my own mind about things	3.19	1.081
C-WEMWBS-12	I’ve been feeling loved	4.18	.866
C-WEMWBS-13	I’ve been interested in new things	2.44	1.136
C-WEMWBS-14	I’ve been feeling cheerful	3.10	1.010

### Descriptive statistics, floor and ceiling effects

**Fig. 1:**
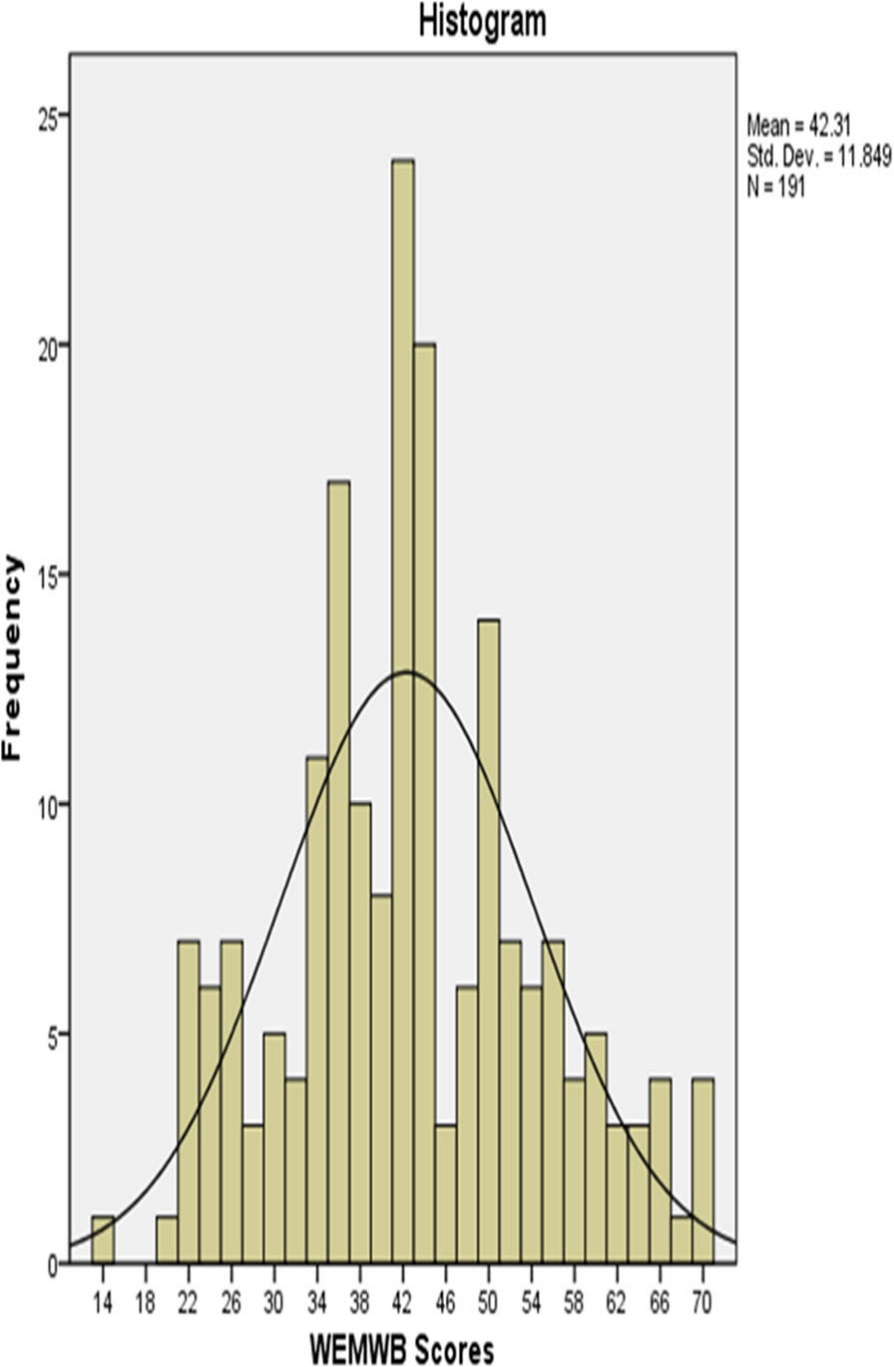
A histogram of the distribution of mean scores: The mean ± SD C-WEMWBS scores were 42.31 ± 11.85 (95% confidence intervals, 40.62–44.01; skewness, 0.176; kurtosis, 0.350) with a median of 42

The mean ± SD of C-WEMWBS scores were 42.31 ± 11.85 (95% confidence intervals, 40.62–44.01; skewness, 0.176; kurtosis, 0.350) with a median of 42(Fig. 1). One-Sample Kolmogorov-Smirnov Test did not display any significant deviation of the response from Normal distribution (Kolmogorov-Smirnov Z = 1.280, *p* > 0.05). All the response categories were used by at least one person for all the scale items. Mean values for the individual items ranged from 2.23 (0.998) for item5 “I have energy to spare” to 4.18 (0.866) for item12 “I’ve been feeling loved” (Table 2).

Among the participants, 31.4 and 41.9% responded “all of the time” for items 9 “I’ve been feeling close to other people” and 12 “I’ve been feeling loved”, meanwhile, 22.5 and 26.7% responded “never” for items 13 “I’ve been interested in new things” and 5 “I’ve had energy to spare” (*n* = 191; Table 3).

**Table 3:** Percentage of floor and ceiling effects of C-WEMWBS (*n* = 191)

Item	Content	Floor effect	Ceiling effect
C-WEMWBS-1	I’ve been feeling optimistic about the future	23(12.0)	15(7.9)
C-WEMWBS-2	I’ve been feeling useful	28(14.7)	15(7.9)
C-WEMWBS-3	I’ve been feeling relaxed	8(4.2)	13(6.8)
C-WEMWBS-4	I’ve been feeling interested in other people	31(16.2)	15(7.9)
C-WEMWBS-5	I’ve had energy to spare	51(26.7)	4(2.1)
C-WEMWBS-6	I’ve been dealing with problems well	17(8.9)	23(12)
C-WEMWBS-7	I’ve been thinking clearly	9(4.7)	23(12)
C-WEMWBS-8	I’ve been feeling good about myself	19(9.9)	17(8.9)
C-WEMWBS-9	I’ve been feeling close to other people	2(1.0)	60(31.4)
C-WEMWBS-10	I’ve been feeling confident	28(14.7)	1(0.5)
C-WEMWBS-11	I’ve been able to make up my own mind about things	8(4.2)	29(15.2)
C-WEMWBS-12	I’ve been feeling loved	2(1.0)	80(41.9)
C-WEMWBS-13	I’ve been interested in new things	43(22.5)	10(5.2)
C-WEMWBS-14	I’ve been feeling cheerful	17(8.9)	13(6.8)

### Factor analysis of dimensionality with principal component analysis

**Fig. 2:**
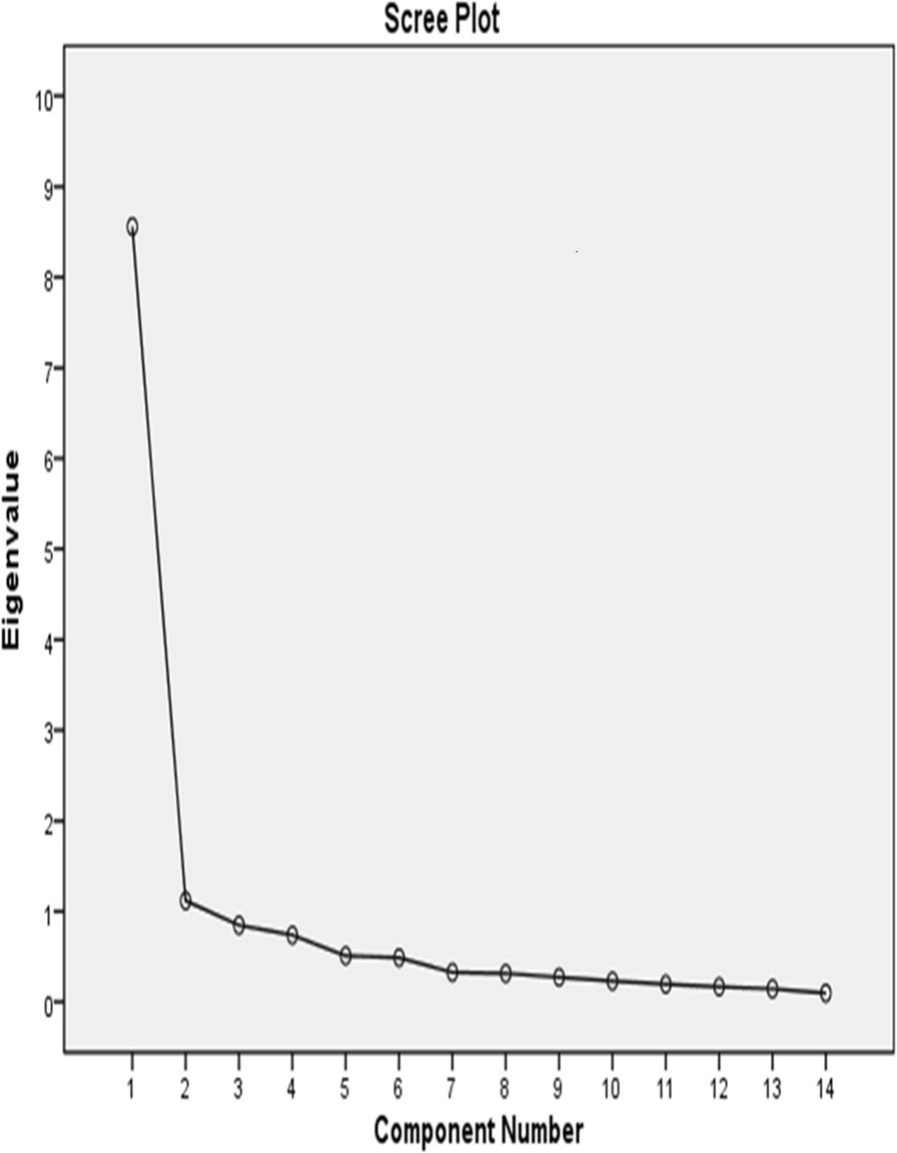
Scree plot for 14-item WEMWBS. Our principal component analysis identified two significant factors with eigenvalues greater than 1, which explained 69.12% of the total variance

We subjected the C-WEMWBS responses to principal component analysis; the correlation matrix showed that all variables had at least one correlation coefficient greater than 0.4. The universal KMO measure was 0.938; individual KMO measures were all greater than 0.9, which indicated appropriate classification. The Bartlett sphericity test was statistically significant (χ^2^ = 2282.83, *df* = 91, *p < 0.05*), indicating that the data were suitable for factor analysis. Our principal component analysis identified two significant factors (Fig. 2) with eigenvalues greater than 1: together, they explained 69.12% of the total variance. The Cronbach alpha value, visual inspection of scree plot, eigenvalues, and interpretability criteria indicated that one component should be retained. The single-component solution explained 61.14% of the whole variance. The total score was obtained by summing all the items. The factor loadings of the rotated component matrix for all 14 items of the C-WEMWBS based on eigenvalues greater than 1 appear in Table 4.

**Table 4:** Factor loadings for the 14 items in the C-WEMWBS in a sample of CHF patients (*n* = 191**)**

Item	Content	Factor loading	
		I	II
C-WEMWBS-1	I’ve been feeling optimistic about the future	.848	
C-WEMWBS-2	I’ve been feeling useful	.902	
C-WEMWBS-3	I’ve been feeling relaxed	.830	
C-WEMWBS-4	I’ve been feeling interested in other people	.763	
C-WEMWBS-5	I’ve had energy to spare	.822	
C-WEMWBS-6	I’ve been dealing with problems well	.881	
C-WEMWBS-7	I’ve been thinking clearly	.802	
C-WEMWBS-8	I’ve been feeling good about myself	.886	
C-WEMWBS-9	I’ve been feeling close to other people	.622	.594
C-WEMWBS-10	I’ve been feeling confident	.543	
C-WEMWBS-11	I’ve been able to make up my own mind about things	.804	
C-WEMWBS-12	I’ve been feeling loved	.451	.734
C-WEMWBS-13	I’ve been interested in new things	.796	
C-WEMWBS-14	I’ve been feeling cheerful	.844	

As shown in Table 4, factor loadings for each item ranged from .451 for item 12 “I’ve been feeling loved” to .902 for item 2 (“I feel useful”). The scree plot is presented in Fig. 2.

### Internal consistency and content validity

The WEMWBS comprises 14 items. The internal reliability coefficient for the single-factor structure of the C-WEMWBS was .948, which was above the recommended lowest limit of .80 [47]. The corrected item correlations for the 14 items were greater than 0.4, thereby supporting the same construct for all items. The item-total statistics for all items appear in Table 5.

**Table 5:** Unstandardized parameter estimates for items of the C-WEMWBS, and model fit estimates for four different one-dimensional models for C-WEMWBS models without correlated error terms, three different one-dimensional models for C-WEMWBS models with correlated error terms (*n* = 191)

Content	Scale Mean if Item Deleted	Scale Variance if Item Deleted	Corrected Item-Total Correlation	Squared Multiple Correlation	Cronbach’s Alpha if Item Deleted
I’ve been feeling optimistic about the future	39.45	119.175	.810	.779	.935
I’ve been feeling useful	39.51	118.220	.872	.846	.934
I’ve been feeling relaxed	39.22	123.141	.789	.656	.937
I’ve been feeling interested in other people	39.71	120.953	.716	.603	.938
I’ve had energy to spare	40.09	122.134	.783	.705	.936
I’ve been dealing with problems well	39.34	118.308	.848	.838	.934
I’ve been thinking clearly	39.21	121.345	.759	.785	.937
I’ve been feeling good about myself	39.42	118.530	.857	.766	.934
I’ve been feeling close to other people	38.39	127.724	.567	.538	.942
I’ve been feeling confident	39.41	118.264	.493	.294	.951
I’ve been able to make up my own mind about things	39.12	121.096	.763	.747	.937
I’ve been feeling loved	38.13	131.599	.405	.331	.945
I’ve been interested in new things	39.87	120.437	.749	.675	.937
I’ve been feeling cheerful	39.21	121.503	.803	.701	.936

The Pearson correlation coefficients for each item-total pair ranged from .405 for item 12 “I’ve been feeling loved” (“I feel loved”) to .872 for item 2 (“I feel useful”). These figures are comparable with those in one studied sample population [10], in which the coefficients ranged from *r* = .50 to *r* = .75.

### Construct validity: external consistency

The Pearson correlation between the C-WEMWBS and WHO-5 was .79 (*P* < .001, *n* = 191) with a confidence interval of .728–.843. This result indicates a strong association between the C-WEMWBS and WHO-5.

### Test-retest reliability

In all, 35 participants completed the retest questionnaires toward assessing the test-retest reliability over a 2-week interval. The intraclass correlation coefficients showed a high test-retest reliability (*r* = 0.925, *P* < .001) for the C-WEMWBS, with a 95% confidence interval of .862–.966.

## Discussion

Building on the previous work of Dong et al. [41], who translated the 14-item WEMWBS and verified the reliability and validity of the scale in a sample of undergraduate nursing trainees, this research adds significant information about measurement model and invariance, reliability, and validity of the C-WEMWBS. The present study apply the C-WEMWBS in evaluating mental well-being in hospitalized patients with CHF. Outcomes showed that the scale may be favorable in predicting psychosocial results in CHF patients, which needs further study to prove. Furthermore, there are some connection between the results of mental well-being measured by the C-WEMWBS and demographic characteristics of patients, such as age, marital status, educational level, self-reported personality. And there are no difference between male patients and female patients which was consistent with the original study of evaluating the WEMWBS [10].

Studies on the WEMWBS in its original and later versions [10, 37–39, 41–44] have demonstrated that it is a useful, reliable instrument for mental well-being, allowing population-level surveys and international comparisons to be performed. The validation of C-WEMWBS will provide the Chinese research society with an instrument prone to explore those elements with a positive impact on people’s mental well-being that might help research to explore ways for empowering individuals, so that they can improve their own quality of life, helping us to evaluate important fields of people’s emotional and social needs concept.

Similar to the results in the United Kingdom validation study of WEMWBS [10], the mean (SD) score for the C-WEMWBS in this study was 42.31 (11.85), which was lower than that for the WEMWBS in general population surveys around the world [24, 37, 38, 42, 44, 54, 55] and among women with urinary incontinence [31]. The C-WEMWBS scores in CHF patients were Normally distributed, which resembled the original scale [10]. Nonetheless, there may be some differences among different groups who could speaking Chinese [41, 56]. C-WEMWBS scores were skewed distributions in undergraduate nursing trainees [41], while the distribution of total scores of Chinese sample living Birmingham was normal with a slight tail towards the lower end [56]. These differences deserve further research.

Unlike with other generally used measuring instruments of mental health, we found that the C-WEMWBS showed that item-5 “I’ve had energy to spare”, and item-13 “I’ve been interested in new things” showed floor effect, while item-9 “I’ve been feeling close to other people” and item-12 “I’ve been feeling loved” displayed ceiling effect. This was not consistent with those of previous validation studies [10, 28, 39, 44, 56]. The results may have been due to the characteristics of the disease: CHF gradually sets limitations on physical activities and becomes a lifelong condition that eventually requires long-term treatment. Such individuals are short of energy and have no interest in new things. Item-9 and item-12 received a positive response: it was as high as 4.18. This could be explained that having a CHF patient in the family would make other family members try their best to provide the greatest help for those individuals owing to the limitations with daily life caused by CHF, they might even hire a housemaid to care for such people.

The C-WEMWBS had good reliability and validity for our sample similar to that of the original instrument. Notably, all the original Scottish [10], Chinese [41], and Spanish samples [44] fit a similar single-factor model, which suggests real deviation in the dominantly positive distribution of mental health traits. This result may reflect differences in the population samples-even in different settings. Therefore, further studies are needed to evaluate the C-WEMWBS in different clinical settings in China as well as with larger populations. Provided that there is no difference between original and Chinese version, potentially effective lines of study open regarding the causes like demographic characteristics and clinical features of these difference.

As in other studies conducted around the world, our analysis confirmed the unidimensional construct of the WEMWBS and its high internal consistency [10, 28, 39, 51, 57]. However, the factor structure was not as defined as in the original validation study [10] and other analyses [28, 39, 51, 57]; this suggests multidimensionality emerging from the exploratory factor analysis. However, according to the researcher’s viewpoint [43] that we could consider the solution as basically unidimensional if the ratio of the first to the second was over 4. And in our analysis, the ratio of the first eigenvalue to the second one was close to 8, and the spectrum of cross loading and the satisfactory additional variance explained by two components (the second component was nearly 1)—together with the lack of a theoretical or explanatory foundation for the factor structure—indicated that a unidimensional model can be assumed.

There was some difference between the item-total correlations identified in the present study and the previous report [56] of Chinese people resident in the United Kingdom. Perhaps the differences are related to differences in the samples: our participants were Chinese-speaking CHF patients, the C-WEMWBS had been translated into Chinese, and cultural differences exist between Eastern and Western countries. Further research needs to determine whether real differences in well-being or cultural issues affect such item-total correlations. Some of our participants indicated that certain words or phrases were difficult to understand and that the clinical setting for administering the questionnaire was a little unsettling for them.

We found that the C-WEMWBS showed a strong internal consistency reliability in this sample group: there was a high Cronbach alpha (0.948), and there were strong internal positive correlations among the item-total scores (ranging from .405 to .872) [58]. The test-retest reliability correlation (intraclass correlation coefficient = 0.925; 95% confidence interval, 0.862–0.966; *n* = 35) was unexpectedly high, indicating that the scale has acceptable stability [59].

The exploratory factor analysis results were consistent with the predicted scale structure, and all factor loadings were equal to or greater than .40. Thus, the C-WEMWBS and original WEMWBS show similar reliability and validity for the whole-scale score. Accordingly, the C-WEMWBS was found to be a reliable, valid scale in our sample of CHF patients. We adopted the WHO-5 as a criterion to investigate the concurrent validity of the C-WEMWBS: we found a significant, positive correlation between the two scales. This result indicates that the C-WEMWBS has good criterion-related validity and suggests that WEMWBS measures a single underlying concept.

CFA showed that the C-WEMWBS has factorial validity. That together with principal component analysis revealing one significant factor with 59.45% of the total variance supports the hypothesized one-dimensional solution. The results from exploratory factor analysis and CFA were consistent, which indicates that the sample data in this study fitted the intrinsic hypothesis.

Our study has several limitations which deserve further research. Firstly, the size of the validation sample was relatively limited and the patients were recruited from a single centre. Secondly, there was short of a golden criterion questionnaire of mental well-being. So the WHO-5 was adopted to evaluate mental well-being to overcome this problem. The current research added evidence of a moderate positive association with WHO-5. Therefore, even acknowledging that complete validity could not be established, the C-WEMWBS shows the resemblance in validity to the original version. Thirdly, the essence of the descriptive, cross-sectional design adopted by the study impeded the evaluation of questionnaire responsiveness. Therefore, it’s necessary to search further evidence about the responsiveness of the C-WEMWBS in an appropriate longitudinal study. Finally, there was only one paper reporting Memorial University of Newfoundland Scale of Happiness in CHF patients [60]. In the following work, we will compare the two scales in the CHF patients to check whether there is any difference.

## Conclusions

To the best of our knowledge, this study is the first attempt to validate the WEMWBS for estimating the mental well-being in a population of CHF patients; thus, our investigation underscores the importance of this scale among the limited number of validated psychometric instruments available. Our participants had lower well-being scores than those reported in general population surveys worldwide. The C-WEMWBS showed good reliability and validity among our participants: it appears to be a reliable, valid instrument for use among Chinese-speaking CHF patients. Further research with larger, more diverse samples is needed to verify the universality of the C-WEMWBS and to examine its applicability to populations with different health statuses.

## Additional file

Additional file 1: The stastical data in the EXCEL incluses results for ANOVA's and their post-hoc tests. (XLS 27 kb)

## Abbreviations

CHF: Chronic heart heart
C-WEMWBS: Chinese version of Warwick-Edinburgh Mental Well-Being Scale
WEMWBS: Warwick-Edinburgh Mental Well-Being Scale
WHO-5: The five-item World Health Organization Well-Being Index
